# Prognostic value of a novel FPR biomarker in patients with surgical stage II and III gastric cancer

**DOI:** 10.18632/oncotarget.20661

**Published:** 2017-09-06

**Authors:** Jing Zhang, Shu-Qi Li, Zhi-Hua Liao, Yu-Huan Jiang, Qing-Gen Chen, Bo Huang, Jing Liu, Yan-Mei Xu, Jin Lin, Hou-Qun Ying, Xiao-Zhong Wang

**Affiliations:** ^1^ Jiangxi Province Key Laboratory of Laboratory Medicine, Department of Clinical Laboratory, The Second Affiliated Hospital of Nanchang University, Nanchang, 330006, China; ^2^ Department of Clinical Laboratory, Jiangxi Provincial Maternal and Child Health Hospital, Nanchang, 330006, China

**Keywords:** inflammation, gastric cancer, prognosis, nomogram

## Abstract

**Background:**

Inflammation and nutrition are two main causes contributing to progression of gastric cancer (GC), and inflammatory biomarker may be presented as its valuable prognostic factor. Thus, this study was carried out to investigate the prognostic significance of preoperative circulating albumin/fibrinogen ratio (AFR), fibrinogen/pre-Albumin ratio (FPR), fibrinogen (Fib), albumin (Alb) and pre-Albumin (pAlb) in surgical GC.

**Materials and Methods:**

Three hundred and sixty surgical stage II and III GC patients from June 2011 to December 2013 were enrolled in this retrospective study. X-tile software, Kaplan–Meier curve and Cox regression model were used to evaluate the prognostic role of them. A predictive nomogram was established to predict prognosis of overall survival (OS), and its accuracy was assessed by concordance index (c-index).

**Results:**

Decreased Alb, pAlb, AFR and elevated FPR were significantly associated with shorter OS. FPR was identified as the most effective prognostic factor to predict 3-year’s OS by time-dependent ROC analysis. A long survival was observed in patients with low level of FPR and the prognosis of stage III FPR-low GC patients undergoing chemotherapy was significantly superior to the patients without the treatment (*P*=0.002). However, no difference of survival was examined in stage II subgroups stratified by FPR and high FRP of stage III patients with or not the treatment of chemotherapy. C-index of nomogram containing FPR (c-index=0.756) was high in comparison with the nomogram without FPR (c-index =0.748).

**Conclusion:**

Preoperative FPR might be a feasible prognostic biomarker in surgical stage II and III GC and it could precisely distinguish stage III patients who appeared to obviously benefit from adjuvant chemotherapy. Meanwhile established nomogram based on clinical parameters and FPR could improve its predictive efficacy.

## INTRODUCTION

Gastric cancer (GC), one of the most common malignancies, is the second most cause of mortality worldwide [1]. Although rapid improvement of surgery and adjuvant treatment in past decade, the prognosis of GC patients remained unsatisfactory owing to recurrence or metastasis after curative resection [2]. Therefore, promising prognostic biomarker which predicted its progression and survival would be helpful for management and treatment in these patients.

It has been well known that inflammation and nutrition are closely associated with progression and survival of GC [3–5]. Anti-inflammatory treatment and nutritional care could prevent cancer progression and improve prognosis of the patients [5–7]. Seo *et al.* reported that preoperative adequate albumin (Alb) and energy intake could improve therapeutic effect of the patients [5]. Kim *et al.* demonstrated that long-term low-dose aspirin intake could reduce susceptibility to GC [7]. Circulating nutritional and inflammatory mediators such as fibrinogen(Fib), Alb and pre-albumin(pAlb) are usually aberrant in these patients. Emerging evidences indicated that high level of plasma Fib were significantly associated with poor clinical outcome of GC patients [8–10], and preoperative low serum pAlb level and hypoalbuminemia were considered to be predictors for shorter overall survival (OS) in GC patients [11, 12]. A recent study reported that circulating albumin to gamma-glutamyltransferase ratio could apparently improve predictive accuracy for OS in resected intrahepatic cholangiocarcinoma patients in comparison with TNM staging systems alone [13]. Thus, we speculated that circulating Alb/Fib ratio (AFR) and Fib/pAlb ratio (FPR), which reflected status of inflammation and nutrition, would be novel inflammatory biomarkers of prognostic prediction for postoperative stage II and III GC patients.

We firstly compared the clinical efficacy of preoperative circulating Fib, Alb, and pAlb, either alone or pooled, for 3 years’ clinical outcome in stage II and III GC patients. Our findings revealed that FPR could independently predict postoperative OS with superior accuracy compared with the other prognostic indicators and select the patients who could benefit from adjuvant chemotherapy. Additionally, a reliable prognostic nomogram based on clinical parameters and FPR could improve its predictive value of OS in the patients.

## RESULTS

### Clinical characteristics of GC patients

Enrolled 360 stage II and III GC patients included 261 male (72.5%) and 99 (27.5%) female and median age was 58 years (ranged from 21 to 86). During 3 years’ following up, 120 (33.3%) patients were confirmed dead and 240 (66.7%) were alive. The postoperative histology results revealed that the majority of the patients were deep invasion (T3/T4) and lymph node metastasis (76.1% and 62.8%, respectively). There were 88 (24.4%) patients with large tumor size (>5cm). Almost half of them had poor differentiated and received adjuvant chemotherapy (55.8% and 69.3%, respectively). The median values of CEA, CA199, Fib, Alb, pAlb, AFR and FPR were 5.59 (0.1-100) ng/ml, 51.81 (0.9-700) U/ml, 3.31 (0.93-6.27) mg/dl, 38.79 (26.21-49.57) g/l, 214.15 (67.3-437.9) mg/l, 12.96 (5.76-43.26), 17.97 (3.03-83.47), respectively. Other details of features are summarized in Table 1.

**Table 1:** Clinical and pathological characteristics in 360 gastric cancer patients

Variables	Categories	Total patients(n=360)
		No. of patients (%)
Gender	Male	261(72.5)
	Female	99(27.5)
Age	year	58.24±11.22
Tobacco	Yes	120(33.3)
	No	240(66.7)
Alcohol	Yes	82(22.8)
	No	278(77.2)
Hypertension	Yes	44(12.2)
	No	316(87.8)
Diabetes	Yes	17(4.7)
	No	343(95.3)
Adjuvant Chemotherapy	Yes	249(69.2)
	No	111(30.8)
Differentiation	well	159(44.2)
	poor	201(55.8)
Tumor stage	II	123(34.2)
	III	237(65.8)
Depth of invasion	T1-T2	86(23.9)
	T3-T4	274(76.1)
Lymph node	N0	134(37.2)
	N1-N3	226(62.8)
Tumor size	≤5cm	272(75.6)
	>5cm	88(24.4)
Fib	mg/dl	3.31(0.93-6.27)
Alb	g/l	38.79(26.21-49.57)
pAlb	mg/l	214.15(67.3-437.9)
FPR		17.97(3.03-83.47)
AFR		12.96(5.76-43.26)
CEA	≤5 ng/ml	309(85.8)
	>5 ng/ml	51(14.2)
CA199	≤37 U/ml	303(84.2)
	>37 U/ml	55(15.3)
	NA	2(0.5%)
3 years’ OS	month	36

Abbreviation: NA: not available; Fib: fibrinogen; Alb: albumin; pAlb: pre-Albumin; AFR: albumin/fibrinogen ratio; FPR: fibrinogen/pre-Albumin ratio (FPR); CEA: carcinoembryonic antigen; FAS: FPR and Alb Score; mFAS: modified FPR and Alb Score; OS: overall survival;

### The optimal thresholds for Fib, Alb, pAlb, AFR and FPR

**Figure 1:**
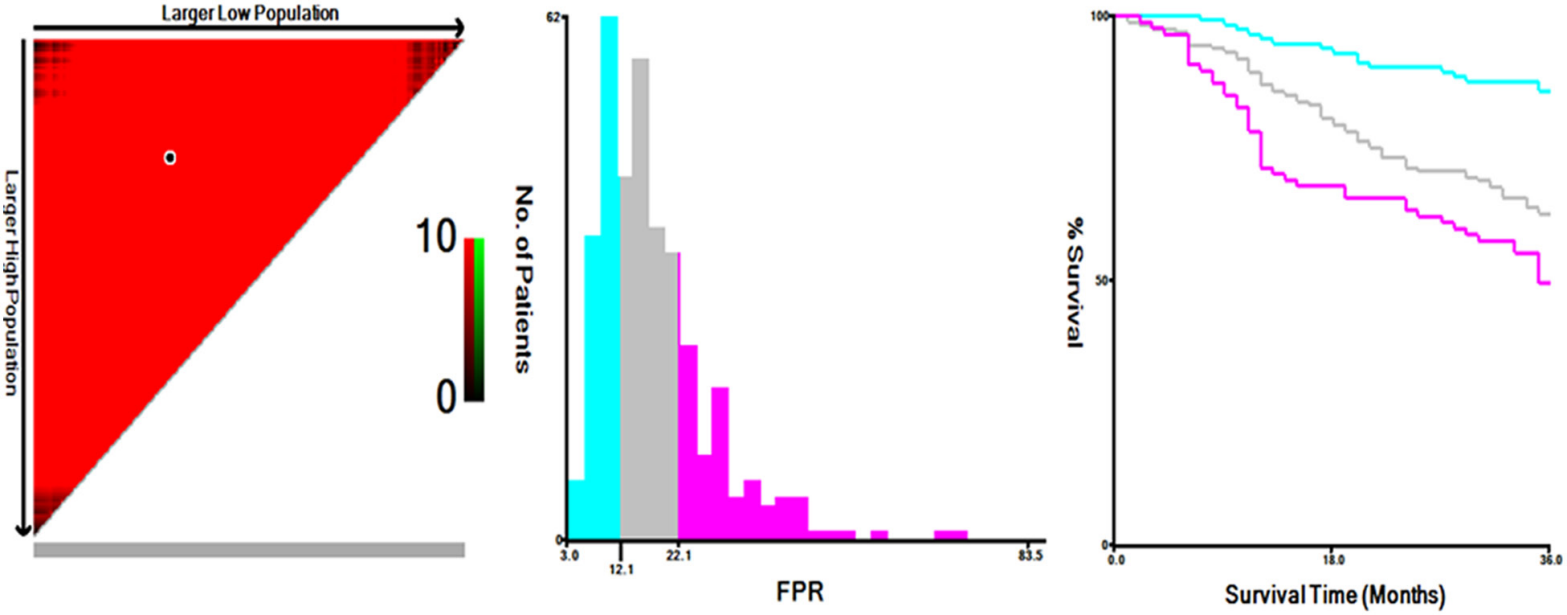
The optimal cut-off of preoperative circulating FPR in 360 surgically resected GC patients using X-tile software The optimal cut-point of FPR ratio in the panels is shown on the histogram and corresponding populations are displayed on the Kaplan–Meier curve.

The optimal cut-points using X-tile program for preoperative circulating Fib, Alb, pAlb, AFR and FPR were 3.3 mg/dl, 37 g/l, 195.9 mg/l, 8.9 and 12.1, respectively (Figure 1 and Supplementary Figure 1). According to the optimal cut-points, enrolled patients were divided into low- and high- groups. The details are shown in Table 2.

**Table 2:** Correlation of preoperative circulating Fib, Alb, pAlb, AFR and FPR with clinicopathological characteristics in 360 GC patients

Characteristics		Patients grouped by		P^*^	Patients grouped by		P^*^	Patients grouped by		P^*^	Patients grouped by		P^*^	Patients grouped by		P^*^
		FPR level (n=360)			AFR level (n=360)			Fib level (n=360)			Alb level (n=360)			pAlb level (n=360)		
		FPR>12.1 FPR≤12.1			AFR>8.9 AFR≤8.9			Fib>3.3 Fib≤3.3 mg/dl			Alb>37 Alb≤37g/l			pAlb>195.9 pAlb≤195.9 mg/l		
Gender	Male	177	84	0.732	204	57	0.062	122	139	0.463	179	82	0.253	154	107	0.049
	Female	69	30		86	13		42	57		74	25		47	52	
Age(years)	≤60	120	86	<0.001	182	24	0.015	70	136	<0.001	162	44	<0.001	127	79	0.010
	>60	126	28		108	46		94	60		91	63		74	80	
Tobacco	Yes	83	37	0.810	90	30	0.060	63	57	0.061	78	42	0.121	64	56	0.499
	No	163	77		200	40		101	139		175	65		137	103	
Alcohol	Yes	55	27	0.780	62	20	0.198	41	41	0.358	52	30	0.122	46	36	0.956
	No	191	87		228	50		123	155		201	77		155	123	
Hypertension	Yes	29	15	0.712	35	9	0.857	24	20	0.201	29	15	0.499	26	18	0.642
	No	217	99		255	61		140	176		224	92		175	141	
Diabetes	Yes	13	4	0.597	9	8	0.003	12	5	0.034	12	5	0.977	6	11	0.081
	No	233	110		281	62		152	191		241	102		195	148	
Chemotherapy	Yes	164	85	0.131	206	43	0.118	109	140	0.310	185	64	0.012	143	106	0.361
	No	82	29		84	27		55	56		68	43		58	53	
Differentiation	well	111	48	0.592	125	34	0.408	71	88	0.760	106	53	0.182	90	69	0.793
	poor	135	66		165	36		93	108		147	54		111	90	
Tumor stage	II	67	56	<0.001	110	13	0.002	40	83	<0.001	100	23	0.001	78	55	0.048
	III	179	58		180	57		124	113		153	84		123	54	
Depth of invasion	T1-T2	45	41	<0.001	78	8	0.006	27	59	0.003	71	15	0.004	56	30	0.047
	T3-T4	201	73		212	62		137	137		182	92		145	129	
Lymph node	N0	83	51	0.045	118	16	0.006	52	82	0.048	105	29	0.010	81	53	0.175
	N1-N3	163	63		172	54		112	114		148	78		120	106	
Tumor size(cm)	≤5	175	97	0.004	232	40	<0.001	112	160	0.003	203	69	0.001	169	103	<0.001
	>5	71	17		58	30		52	36		50	38		32	56	
CEA(ng/ml)	≤5	203	106	0.008	255	54	0.020	136	173	0.143	218	91	0.781	178	131	0.096
	>5	43	8		35	16		28	23		35	16		23	28	
CA199(U/ml)	≤37	201	102	0.045	244	59	0.823	134	169	0.244	214	89	0.818	175	129	0.163
	>37	44	11		45	10		29	26		38	17		26	29	
OS	alive	143	97	<0.001	208	32	<0.001	91	149	<0.001	185	55	<0.001	157	83	<0.001
	dead	103	17		38	38		73	47		68	52		44	76	

### The correlation of Fib, Alb, pAlb, AFR and FPR with the clinical parameters

**Figure 2:**
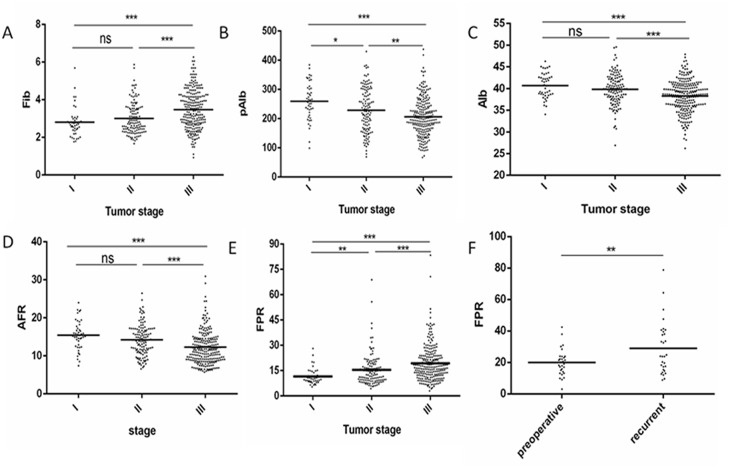
The relationship between tumor stage and Fib, pAlb, Alb, AFR, FPR in 360patients with GC and comparison of FPR in preoperative and recurrent 30 GC patients **(A)** Fib; **(B)** pAlb; **(C)** Alb; **(D)** AFR; **(E)** FPR; **(F)** comparison of FPR in preoperative and recurrent patients. *P<0.05, **P<0.01, ***P<0.001, ns: not significant.

In order to investigate associations of these factors with tumor stage, 44 stage I GC patients were enrolled in our study. We compared the high groups and low groups for these indicators and increased Fib, FPR and deceased Alb, pAlb and AFR were positively correlated with age (more than 60 years), tumor size (larger than 5cm), tumor stage (III), depth of invasion depth (T3-T4), lymph node metastasis (N1-N3) and poor OS (all *P*<0.001) (Table 2 and Figure 2). Compared with 30 preoperative patients, higher FPR were in the patients with recurrent GC (*P*=0.001) (Figure 2F). Besides, no significant association was observed among alcohol, tobacco, hypertension, diabetes, tumor differentiation and adjuvant chemotherapy in two groups.

### The association between baseline characteristics and clinical prognosis

Kaplan–Meier survival curve and log-rank test were performed to investigate the association between pathological data and postoperative 3-year survival time. The 3 years’ OS curves according to Fib, pAlb, AFR and FPR were shown in Supplementary Figure 2. Both Kaplan–Meier curves and univariate analysis showed that sex, tobacco, alcohol, hypertension and diabetes were not significantly associated with OS (*P*>0.05), while patients with older age (HR=1.541, *P*=0.018), worse differentiation (HR=1.890, *P*=0.001), larger tumor size (HR=2.580, *P*<0.001), deeper invasion (HR=6.238, *P*<0.001), higher tumor stage (HR=5.872, *P*<0.001), CEA (HR=2.250, *P*<0.001), CA199 (HR=1.932, *P*=0.003), Fib (HR=2.142, *P*<0.001) and FPR (HR=3.373, *P*<0.001), lower Alb (HR=2.140, *P*<0.001), pAlb (HR=2.672, *P*<0.001), AFR (HR=2.343, *P*<0.001), worse tumor differentiation (HR=1.890, *P*=0.001) and more lymph node metastases (HR=3.874, *P*<0.001) were significant prognostic factors for worse OS (Table 3). Multivariate analysis showed that not only worse differentiation (adjusted HR=1.774, *P*=0.005), larger tumor size (adjusted HR=1.930, *P*=0.001) and more lymph node metastases (adjusted HR=2.201, *P*=0.009), but also lower Alb (adjusted HR=1.614, *P*=0.014), pAlb (adjusted HR=2.111, *P*<0.001), AFR (adjusted HR=1.540, *P*=0.044) and higher CEA (adjusted HR=1.739, *P*=0.013), FPR (adjusted HR=2.325, *P*=0.002) were identified as independent prognostic factors for shorter OS, but age, Fib and CA199 were not (*P*>0.05) (Table 3).

**Table 3:** Univariate and multivariate analyses of prognostic factors for 3 years’ OS by Cox regression model

Variables	Overall survival					
	Univariate analysis			Multivariate analysis		
	HR	95% CI	*P*	HR	(95% CI)	*P*
Sex (male)	11.051	(0.701-1.574)	0.811	-		-
Age (>60 years)	1.541	(1.077-2.205)	**0.018**	1.328	(0.905-1.948)	0.147
Tobacco (yes)	1.020	(0.698-1.490)	0.920	-		**-**
Alcohol (yes)	1.167	(0.772-1.765)	0.463	-		-
Hypertension (yes)	1.345	(0.815-2.220)	0.247	-		-
Diabetes (yes)	1.483	(0.724-3.309)	0.281	-		-
Chemotherapy (no)	1.493	(1.032-2.161)	**0.034**	1.682	(1.136-2.488)	**0.009**
Differentiation (poor)	1.890	(1.289-2.770)	**0.001**	1.774	(1.193-2.639)	**0.005**
Tumor stage (III)	5.872	(3.233-10.67)	**<0.001**	5.006	(2.712-9.241)	**<0.001**
Depth of invasion (T3-T4)	6.238	(2.906-13.39)	**<0.001**	2.293	(0.917-5.738)	0.076
lymph node (N1-N3)	3.874	(2.372-6.328)	**<0.001**	2.088	(1.202-3.626)	**0.009**
Tumor size(>5cm)	2.580	(1.790-3.720)	**<0.001**	1.930	(1.326-2.808)	**0.001**
CEA (>5 ng/ml)	2.250	(1.465-3.456)	**<0.001**	1.739	(1.123-2.694)	**0.013**
CA199 (>37U/ml)	1.932	(1.258-2.968)	**0.003**	1.119	(0.686-1.824)	0.653
Fib (>3.0 mg/dl)	2.142	(1.484-3.091)	**<0.001**	1.463	(0.996-2.149)	0.052
Alb (≤36.4 g/l)	2.140	(1.490-3.072)	**<0.001**	1.614	(1.103-2.361)	**0.014**
pAlb (≤194.1 mg/l)	2.672	(1.842-3.875)	**<0.001**	2.111	(1.437-3.100)	**<0.001**
AFR (≤8.9)	2.343	(1.594-3.445)	**<0.001**	1.540	(1.013-2.343)	**0.044**
FPR (>12.1)	3.373	(2.018-5.636)	**<0.001**	2.325	(1.372-3.940)	**0.002**

Abbreviation: HR: hazard ratio; CI: confidence interval; Fib: fibrinogen; Alb: albumin; pAlb: pre-Albumin; AFR: albumin/fibrinogen ratio; FPR: fibrinogen/pre-Albumin ratio (FPR); CEA: carcinoembryonic antigen; HR (95%) was adjusted by sex, age, alcohol, tobacco, hypertension, diabetes, chemotherapy, tumor size, tumor grade, tumor stage, CEA and CA199.

### Time-dependent ROC analysis

**Figure 3:**
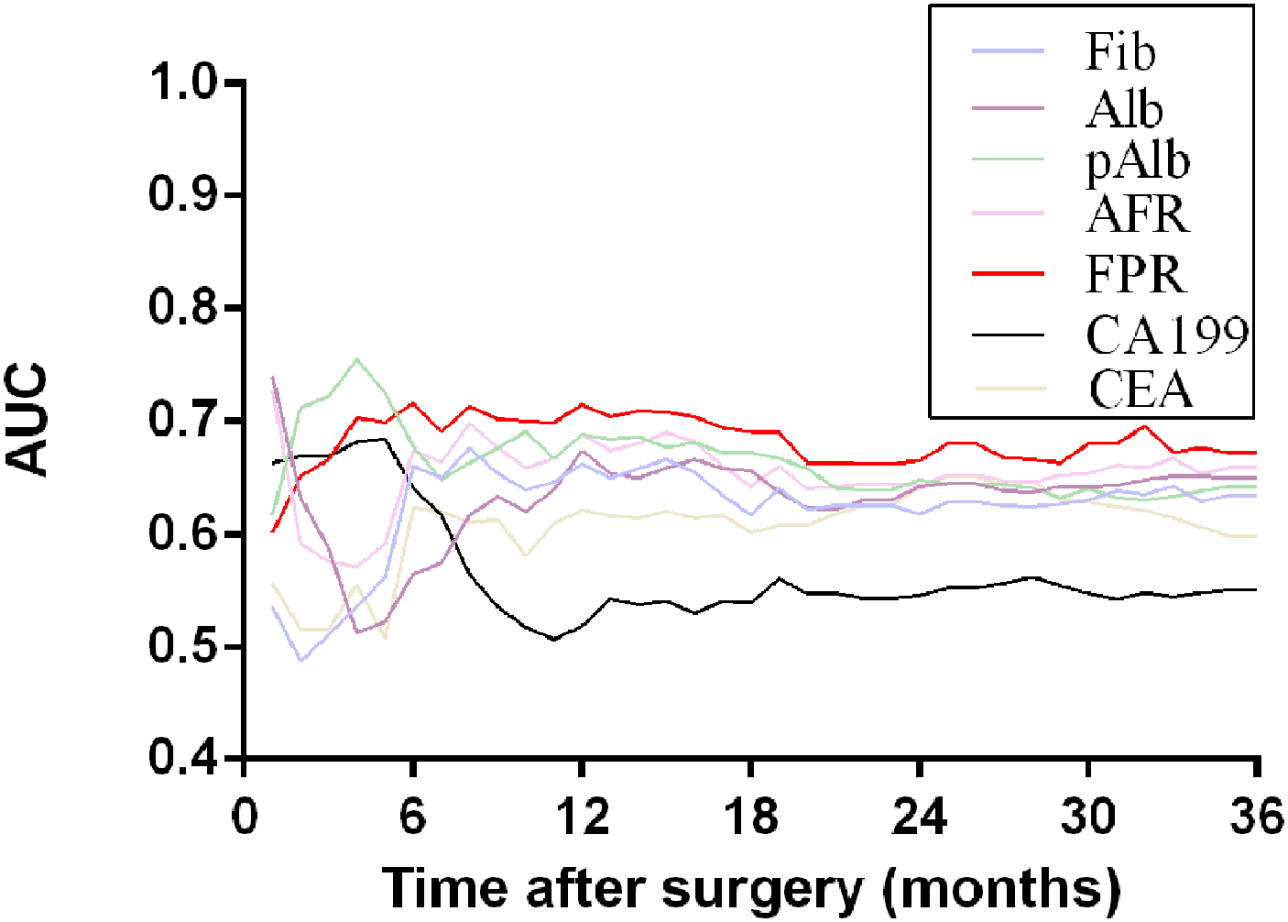
Time-dependent ROC analysis of preoperative circulating Fib, Alb, pAlb, AFR, FPR, CA199 and CEA for clinical outcome of 360 GC patients

To further evaluate the prognostic value of inflammation-based prognostic factors, time-dependent ROC analysis was performed. The result of time-dependent ROC analysis presented that the lower area under the receiver operating characteristic curve (AUC) for FPR in the early period (<6 months) and the higher AUC therefore (>6 months) among these prognostic indicators including Fib, Alb, pAlb, AFR, CEA and CA199 (Figure 3).

### FPR and clinical adjuvant chemotherapy

**Figure 4:**
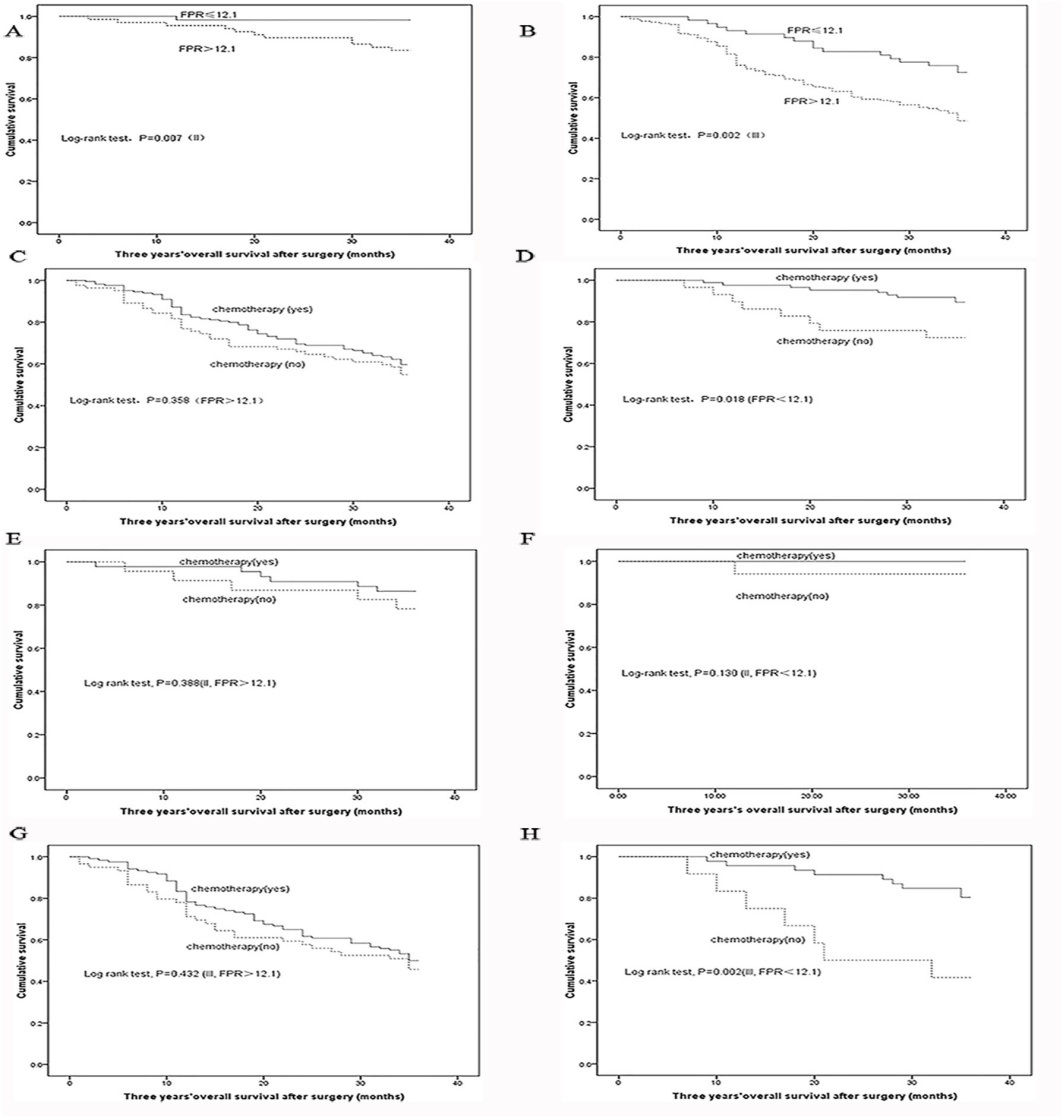
Kaplan–Meier curves analysis in each subgroup **(A)** stage II patient; **(B)** stage III patient; **(C)** high FPR subgroup; **(D)** low FPR subgroup; **(E)** FPR-high stage II subgroup; **(F)** FPR-low stage II subgroup; **(G)** FPR-high stage III subgroup; **(H)** FPR-low stage III subgroup.

We compared the prognosis of stage II and III GC patients receiving or not adjuvant chemotherapy in the subgroups stratified by FPR. Survivals of stage II and III GC patients were significantly longer in low FPR subgroup than them in high FPR subgroup (*P*=0.007 and *P*=0.002, respectively). Low level of FPR (adjusted HR=5.851, 95%CI=2.147-15.949) were significantly associated with reduced survival in the III stage patients without chemotherapy comparing to the patients undergoing chemotherapy. However, no difference of survival was examined in stage II subgroups stratified by FPR and high FRP of stage III subgroup receiving or not the treatment of adjuvant chemotherapy (Table 4 and Figure 4).

**Table 4:** Univariate and multivariate analyses of high/low FPR for chemotherapy by Cox regression model

Variables	Chemotherapy	Univariate analysis			Multivariate analysis		
		HR	(95% CI)	*P*	HR	(95% CI)	*P*
FPR(>12.1)	yes	1			-		
	no	1.205	0.806-1.803	0.363	-		
FPR(≤12.1)	yes	1			1		
	no	2.990	1.153-7.754	0.024	5.851	2.147-15.949	0.001

Abbreviation: HR: hazard ratio; CI: confidence interval; FPR: fibrinogen/pre-Albumin ratio; HR (95%) was adjusted by sex, age, alcohol, tobacco, hypertension, diabetes, tumor size, tumor grade, tumor stage, CEA and CA199.

### Prognostic nomogram for 3-year overall survival

**Figure 5:**
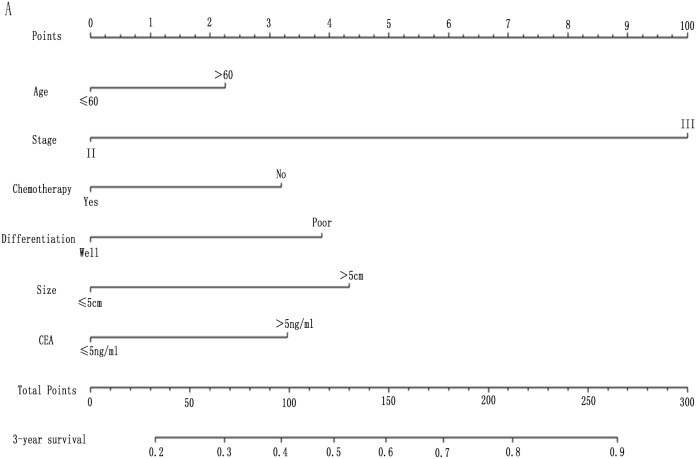

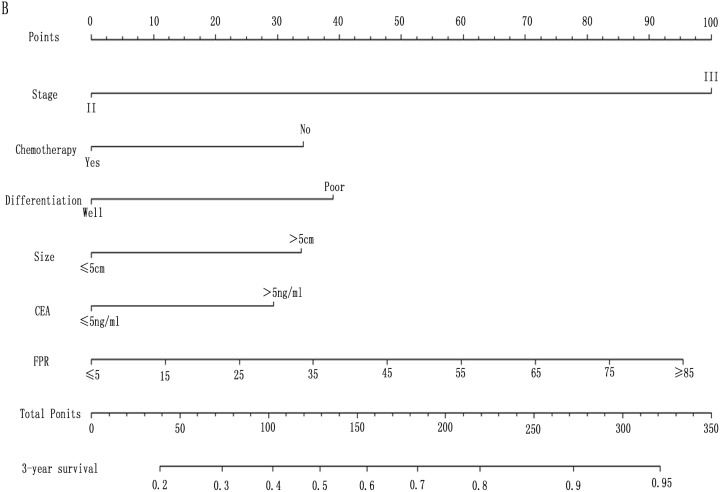
Postoperative nomogram estimated by clinical characteristics and FPR for 3-years’ OS in 360 GC patients **(A)** without FPR; **(B)** with FPR.

To predict the survival of stage II and III GC patients underwent surgical resection, prognostic nomograms were established using all the significantly independent indicators for OS (Figure 5A). The nomogram with FPR (c-index: 0.756) was more accurate than that without FPR (c-index: 0.748) in prediction of 3-year OS after initial surgery (Figure 5B).

## DISCUSSION

Most of the GC patients are diagnosed in an advanced stage and the survival rates of them are relatively low, therefore, promising prognostic biomarkers that enable to identify the patients who could obviously benefit from chemotherapy and predict survival of them are crucial [14, 15]. In this study, we found that evaluated FPR was significantly associated with T3-4 invasion, node metastasis and larger tumor size and was superior to other biomarkers to independently predict poor survival both within stage II-III, II and III subgroups; moreover, clinical outcome of III stage patients with low FPR appeared to obviously benefit from adjuvant chemotherapy in comparison with high FPR stage III patients, and the biomarker could improve the predicted efficacy of nomogram for stage II-III GC.

To date, some researchers have reported that high level of Fib, low level of Alb and pAlb were recognized as important prognostic factors influencing cancer progression [10, 11, 16], which were consistent with our findings. Due to few patients died from the disease within 6 months after surgical resection, low AUC of FPR was observed in the early period, and the AUC was gradually increased and higher than the other biomarkers, indicating that FPR was superior to these biomarkers to apparently improve predictive efficacy of prognosis within II-III stage GC patients. In addition, it could precisely classify stage III GC patients who appeared to benefit from adjuvant chemotherapy obviously. The following causes might be accounted for our findings. Firstly, it had been shown that Fib acts as a bridging molecule between GC cells and surrounding microenvironment. Adams *et al.* demonstrated that it as a ligand for integrin and intercellular adhesion molecule presented on malignant cell surface to mediate coagulation, inflammation and immunity [17]. Secondly, Fib enhanced b3-integrin-mediated vascular endothelial adhesion of platelets to tumor cells, and platelets in turn promoted more Fib to aggregate around tumor cells by forming thrombin. They facilitated each other to protect tumor cells escaping from cytotoxicity of nature killer cells [18]. Palumbo *et al.* reported that lymphatic metastasis, but not primary tumor growth or angiogenesis, was diminished in fibrinogen-deficient mice, suggesting that Fib was a critical determinant of the metastatic potential by impeding elimination of tumor cell by natural killer cell [19, 20]. Thirdly, serum Alb was one of the most widely used markers for reflecting nutritional status and hypoproteinmia was reported as a crucial parameter of malnutrition and directly influenced prognosis of GC; low levels of Alb and pAlb levels have an impact on determinant of immune responses and malnutrition, which could impair immune system defending against GC [21, 22].

This study, to best of our knowledge, is the first to investigate prognostic role of AFR and FPR in GC. Certain advantages and limitations should address to explain our results. To some extent, hypoalbuminemia has been considered to be an inflammatory indicator in GC, rather than only a factor indicates malnutrition [4]. Therefore, single clinical blood marker is limited and unstable to predict prognosis of GC. Our results did firstly find that FPR is a superior prognostic indicator compared to Fib, Alb, or pAlb alone, for they reflected not only inflammation but also nutritional status of GC patients. Besides, circulating Fib to pAlb ratio will expand prognostic range to avoid a single indicator causing false negative or positive results. Finally, we figured out the visual nomogram based on FPR, which could predict prognosis in postoperative stage II and III GC patients within 3 years more accurately. Therefore, preoperative calculation of FPR may help to predict 3 years’ OS in surgical GC patients. However, we acknowledge some potential limitations in our study. Since the results of our study may be affected by a short follow-up period, single-institution design and a small sample size retrospective study, larger patients with GC are required to confirm our findings.

## MATERIALS AND METHODS

### Patients

Three hundred and sixty newly diagnosed stage II and III GC patients were included in this retrospective study and all of them underwent surgical resection from June 2011 to December 2013 at the Second Affiliated Hospital of Nanchang University. The diagnostic criteria for GC were according to the seventh edition of tumor-nodes-metastasis (TNM) staging system [23]. In the contrary, patients were excluded as follows: 1) all patients had infection or inflammation-related diseases for nearly one month, autoimmune diseases and blood diseases; 2) patients who received preoperative anti-inflammatory or anticancer therapy; 3) patients with abnormal liver function, mixed cancers and distant metastasis; 4) absent data regarding preoperative prognostic biomarkers. The study was approved by the Ethical Committee of the Second Affiliated Hospital of Nanchang University.

### Data collection and laboratory detection

Through patients’ medical record and pathological report, we gathered data including age, sex, personal history and postoperative clinical and pathological characteristics. All peripheral blood samples were collected at 7:30 to 9:30 am within three days before surgical operation. Plasma and serum samples were centrifuged at 3000g for 5 min. Plasma Fib concentration were detected using Clauss method by SYSMEX CA-7000 machine (Sysmex, Tokyo, Japan), its inter- and intra-batch coefficient of variation (CV) of the kit were 4.41% and 3.66%, respectively. Bromocresol green, immune turbidimetric and electrochemiluminescence methods were used to detect serum Alb, pAlb, CA199 and CEA using OLYMPUS AU5400 machine(Beckman Coulter, Tokyo, Japan) and COBAS e411 (Roche, Basel, Switzerland), respectively. The inter- and intra-batch CVs of the kits were 3.17% and 1.83%, 3.09% and 2.76%, 3.32% and 3.25%, 3.48% and 3.26%, respectively. In each batch, blinded quality controlled samples were included, and all the markers were measured triplicate in all plasma samples.

### Follow-up

After surgery, all stage II and III GC patients were followed up regularly until December 31^th^ 2016 (every 6 months up to 3th year by telephone). For drop-out patients, the date was obtained by outpatient medical records. Overall survival (OS) was measured from the date of operation to death from any causes or last following-up.

### Statistical analysis

The optimal cut-off levels of prognostic factors were determined by X-tile software. Chi-square test and Mann–Whitney *U* or Kruskal–Wallis test were used to analyze categorical variables and continuous variables with non-normal distributions, respectively. Kaplan–Meier survival curve was applied for survival analysis and the differences in survival rate were performed by the log-rank test. Hazards ratio (HR) for death was estimated with a Cox proportional hazards model. Prognostic nomogram, Harrell’s concordance index (c-index) and Time-dependent ROC were analyzed by the rms and survivalROC packages, respectively. Statistical analyses were carried out using SPSS 19.0 software (IBM Corp, Armonk, NY, USA) and R 3.0.3 software (Institute for Statistics and Mathematics, Vienna, Austria). *P*<0.05 was indicated statistically difference.

## CONCLUSIONS

It is worth emphasizing that preoperative FPR is more compelling in predicting three years’ OS in surgical stage II and III GC than Fib or pAlb alone and adjuvant chemotherapy might be more beneficial to FPR-low stage III GC patients. Due to survival heterogeneity of GC patients, larger cohort prospective studies, especially prospective multicenter clinical trials, are warranted to further validate the results.

## SUPPLEMENTARY MATERIALS FIGURES


