# Supplementary material for: Prognostic value of a novel FPR biomarker in patients with surgical stage II and III gastric cancer
Source: Oncotarget. 2017 Sep 6;8(43):75195–205. doi: 10.18632/oncotarget.20661 (PMC5650412; doi:10.18632/oncotarget.20661)
Supplement: Supplementary file 1 [file oncotarget-08-75195-s001.pdf]

Prognostic value of a novel FPR biomarker in patients with surgical stage II and III gastric cancer

SUPPLEMENTARY MATERIALS

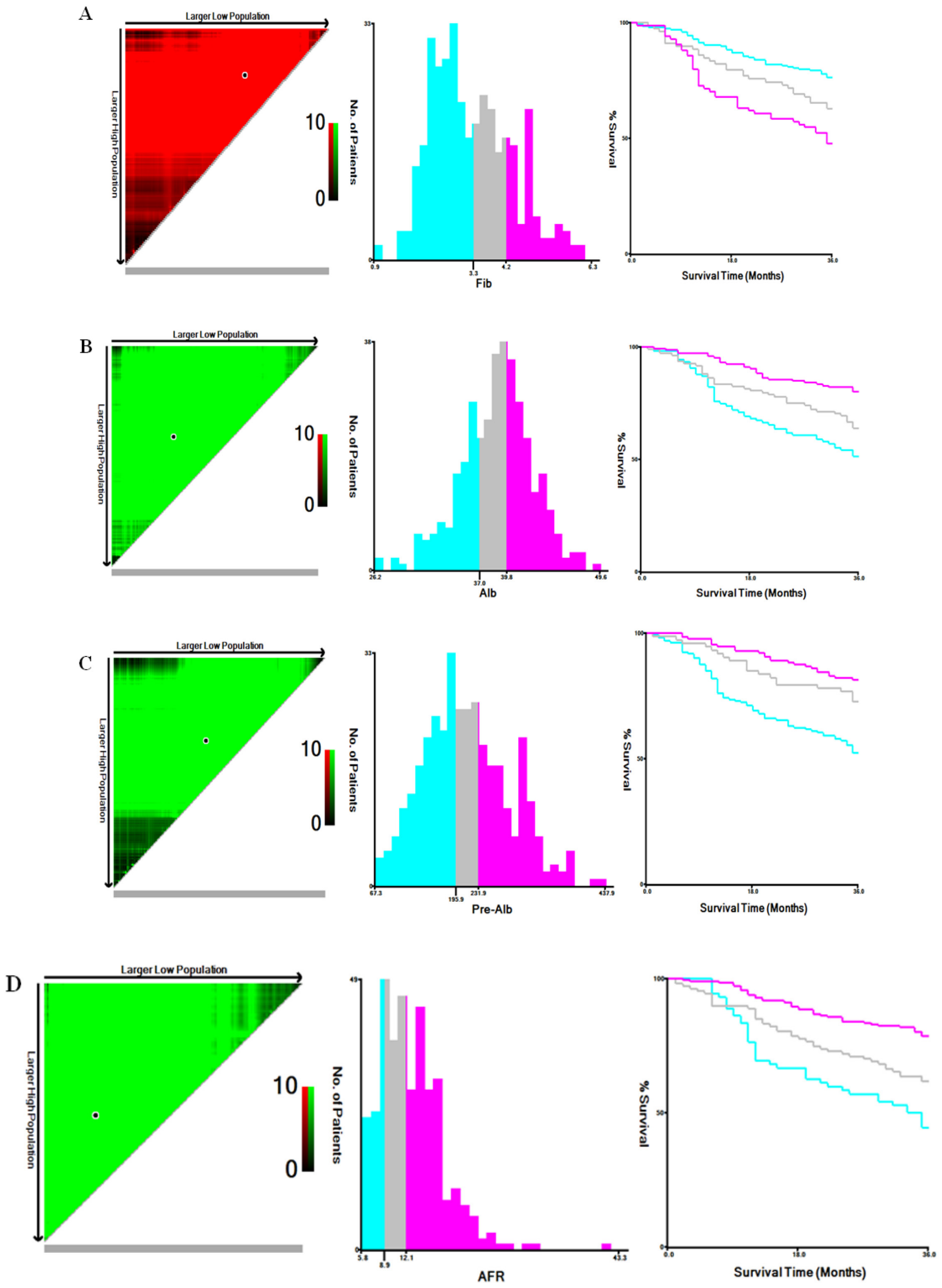

Supplementary Figure 1: The optimal cut-off of preoperative circulating Fib, Alb pAlb, and Alb/Fib in 360 surgically resected gastric cancer patients using X-tile software. **(A)** Fib, **(B)** Alb, **(C)** pre-Alb, **(D)** AFR.

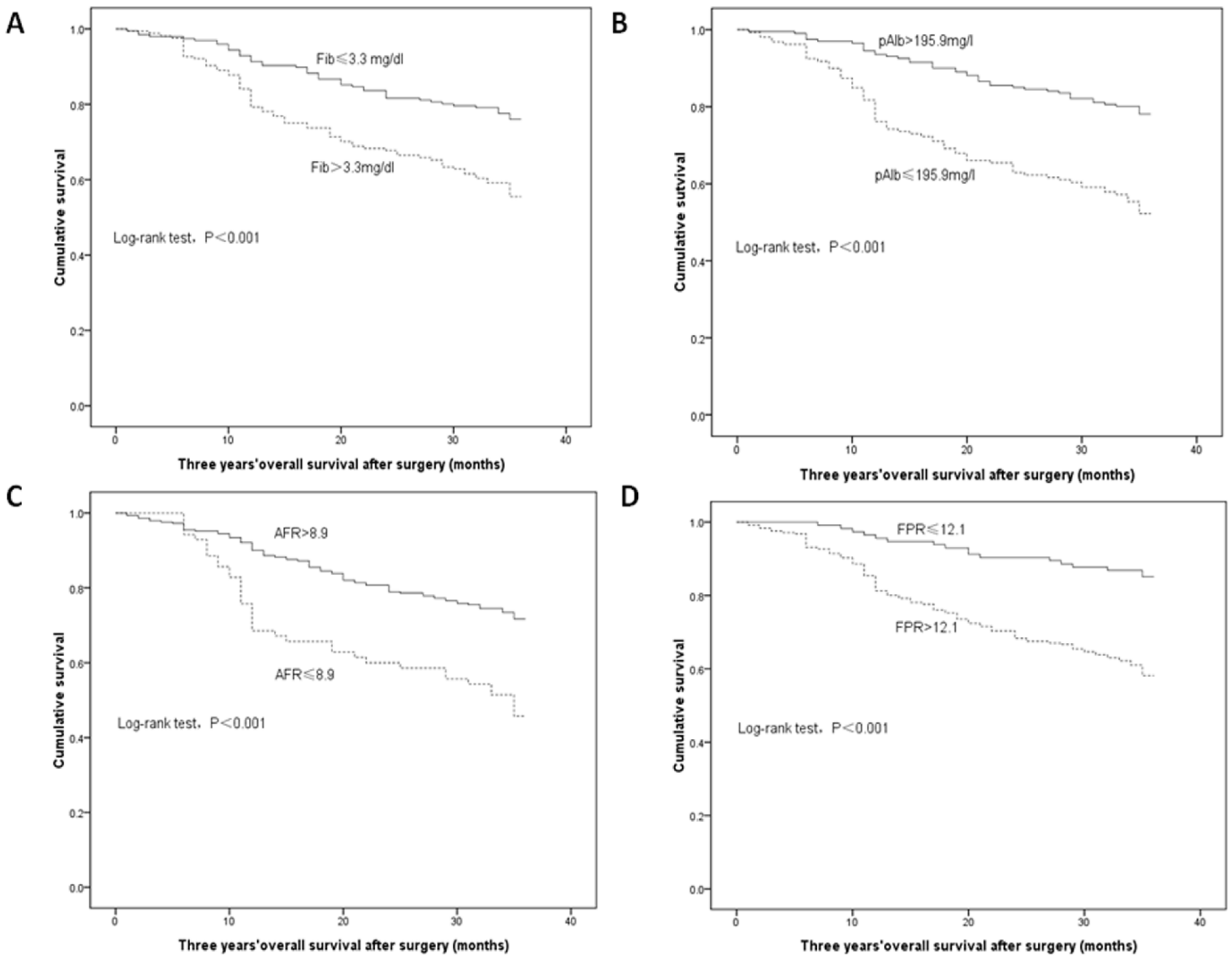

Supplementary Figure 2: Kaplan-Meier curves analysis of Fib, pAlb, AFR and FPR for three years' OS in 360 GC patients. **(A)** Fib; **(B)** pAlb; **(C)** AFR; **(D)** FPR.
